# Prevalence and predictors of overweight and obesity in Brazilian immigrants in Massachusetts

**DOI:** 10.1186/s12889-020-8144-8

**Published:** 2020-01-10

**Authors:** Rachel A. Klabunde, Felippe Lazar Neto, Andressa Louzada, Ricardo Faé de Moura, Fernando Morelli Calixto, Goodarz Danaei, Marcia C. Castro

**Affiliations:** 1 Harvard T.H. Chan School of Public Health, Department of Global Health & Population, 665 Huntington Avenue, Bldg. 1, Boston, MA 02215 USA; 2 Faculty of Medical Sciences, University of São Paulo, São Paulo, Brazil; 3 Santa Casa of São Paulo, Faculty of Medical Sciences, São Paulo, Brazil; 4 Harvard T.H. Chan School of Public Health, Department of Epidemiology, Boston, MA USA

**Keywords:** Immigrant health, Brazilians, USA, Overweight, Obesity, Consumption, Meat, Soda, Fruits, Vegetables

## Abstract

**Background:**

Overweight and obesity are important risk factors for chronic non-communicable diseases, and their prevalence is on the rise worldwide. This study seeks to describe the prevalence and predictors of overweight and obesity in Brazilian immigrants living in Massachusetts, United States of America (USA).

**Methods:**

Modeled after a survey on behavioral risk factors for chronic disease conducted annually in Brazil (*Vigilância de Fatores de Risco e Proteção para Doenças Crônicas por Inquérito Telefônico*: Vigitel), Brazilian immigrants aged 18+ (*n* = 361) were surveyed between December 2013 and March 2014. Information was obtained from consenting participants regarding their demographic characteristics, physical activity, dietary and lifestyle habits, and other behavioral risk factors. Weight status was estimated from body mass index (BMI), calculated from self-reported height and weight data. Participants were categorized as overweight/obese if their BMI was ≥25; overweight and obese categories were combined to ensure appropriate sample size. Prevalence of overweight/obesity was estimated using STATA, and significant predictors were identified via multi-variable logistic regression. Odds ratio (OR), 95% confidence intervals (95% CI) and *p*-values were determined.

**Results:**

The overall prevalence of overweight/obesity in the sample was 47.6%. Significant predictors of overweight and obesity were gender (men OR 2.30, 95% CI: 1.10, 3.78; women are comparison group), working in the 3 months prior to the survey (OR 2.90, 95% CI: 1.01, 8.30), and longer duration living in the USA (OR per additional year 1.06, 95% CI: 1.02, 1.11). Significant dietary predictors of overweight/obesity included 5 or more days per week of consumption of red meat (OR red meat 3.70, 95% CI: 1.47, 9.26) or of sweetened beverages, like soft drinks also known as soda (OR soda 2.40, 95% CI: 1.00, 5.78) compared with less frequent consumption of these foods.

**Conclusions:**

This study suggests that long duration of time lived in the USA increases odds of overweight and obesity for Brazilian immigrants living in Massachusetts. Efforts to curb increases in overweight and obesity in this population should focus not only on the men and those who work but also the women. Possible intervention measures should target soda (soft drink) and red meat consumption in Brazilian immigrants.

## Background

The epidemic of overweight and obesity is at the forefront of global public health agendas. High body mass index (BMI) was ranked as the fifth-leading risk factor for death in 2016, globally [1]. In the USA, 39.8% of adults aged 20 or older were obese in 2016 (BMI ≥ 30) [2]. However, newly-arrived immigrants in the USA have lower levels of obesity, and better overall health, than the native-born population on arrival to the country [3–6]. This “healthy immigrant” phenomenon tends to fade over time. Immigrants who have lived in the USA for at least 15 years have overweight and obesity levels approaching those of USA-born adults [4, 7], and a positive association is generally found between duration of residence in the USA and BMI [4, 7–10]. Acculturation, or immigrant acceptance and adoption of behaviors and practices common in the host culture, is a common explanation for these findings [8, 11–14]. Dietary acculturation, including increased consumption of fast food and soft drinks in the USA compared with the native country, is one potential mechanism for the observed relationship between time since immigration and increased weight [10, 15].

Much of the literature on immigrant health and acculturation in the USA has excluded an important group: Brazilian immigrants. The USA is home to the largest population of Brazilian immigrants in the world, and of the states, Massachusetts (MA) has the second-largest population of Brazilian immigrants after Florida [16]. According to estimates from the American Community Survey (ACS), 336,000 foreign-born Brazilians lived in the USA in 2014, with 61,000 of these living in MA [17]. However, these data could be significant underestimates, as approximately 70% of the Brazilian immigrant population in the USA are undocumented, making them less likely to be counted by surveys like the ACS [18]. Additionally, given the linguistic and cultural uniqueness of Brazilians among South Americans, they are less likely to be correctly counted in the U.S. Census (they are not considered to be “Hispanic”, as Spanish is not their mother tongue) [18]. Few studies exist on the health status or risk factor profile of Brazilian immigrants in the USA [16, 18, 19].

To fill this gap in the literature, this study seeks to estimate the prevalence of overweight and obesity in the Brazilian immigrant population living in MA, compare this prevalence with national estimates for the USA and Brazil, and identify predictors of overweight and obesity in this population.

## Methods

### Participants and data collection

To investigate the health status of Brazilian immigrants living in MA, a cross-sectional survey was conducted between December 2013 and March 2014. The survey was administered in person, with a convenience sample of 520 individuals in two settings. Sample size was determined based on researcher capacity at the time of survey. Most respondents (85%) answered the survey while seeking services at the Consulate General of Brazil in Boston. The rest completed the survey following religious services for the Brazilian immigrant community at MA churches in Cambridge, Framingham, and Somerville, as these were known gathering places for the Brazilian immigrant community in the greater Boston area. The Consulate General of Brazil in Boston, MA was selected as the primary location for survey administration given the wide variety of services provided to Brazilians there (including issuing of documents, voting assistance, vital registration services, etc.), helping to ensure sampling of immigrants of varying age, socioeconomic status, place of residence within MA, and other demographic characteristics.

To be eligible to participate in the survey, a person had to be (a) born in Brazil; (b) 18 years old or older; and (c) living in MA at the time of survey. Informed consent was obtained from all individual participants included in the study. Consent was performed in Portuguese with the assistance of Brazilian medical students, and following consent, participants responded individually to the written questionnaire.

### Survey, health and lifestyle measures

The survey was modelled after Vigitel (an annual, telephone risk factor surveillance survey used in Brazil: Vigilância de Fatores de Risco e Proteção para Doenças Crônicas por Inquérito Telefônico) [19]. While the original Vigitel survey has been used in Brazil since 2006 [19], the present survey was the first of its kind to survey chronic disease risk factors and to specifically sample Brazilians living in MA. It was designed to allow direct comparison with the Vigitel survey [19] in terms of risk and protective factors, with the addition of questions relevant to immigrants. Our survey also included time lived in the USA and in the current city of residence, English proficiency, changes in dietary patterns after immigrating, monthly income categories, as well as standardized questions to evaluate depression [20].

Height and weight were self-reported. Lifestyle habits assessed in the survey included physical activity, smoking habits, alcohol consumption, amount of time spent watching daily TV, and dietary habits. Specifically, respondents were asked about their frequency of consumption of a variety of foods (categorically, in days per week). Portion sizes of food consumption were not addressed in the survey. Respondents were also asked whether their consumption of these items and physical activity level had increased, decreased, or stayed the same compared with their habits when living in Brazil.

### Data analysis

Self-reported height and weight were used to calculate BMI, and participants were classified as overweight/obese if they had a BMI ≥ 25 kg/m2 [21]. To be eligible for inclusion in this analysis, respondents must be Brazilian immigrants, have reported measures of height and weight, as well as all relevant covariates, selected based on a priori knowledge and relevant literature on overweight and obesity [22–25]. Pregnant women were excluded from the analysis. Varying degrees of missing data were present across these covariates. To examine whether missing data was associated with overweight and obesity, a “missing” category was created for all covariates with > 5% missing, and univariate logistic regression models of overweight and obesity on variables with missing data were run. Missing data was not statistically significantly associated with overweight and obesity; therefore, a complete case analysis of these data was performed (final sample *n* = 361).

2Descriptive statistics of the sample were generated. Prevalence of overweight and obesity in the sample, as well as national prevalence estimates from the 2014 National Health Interview Survey (NHIS) [26] and the Brazil 2006–2015 Vigitel surveys [19] were age-standardized to the immigrant sample age distribution to allow for easy comparison. To assess predictors of overweight and obesity in this population, multivariable logistic regression of overweight/obese status on relevant covariates were run. Relevant covariates included in the model were selected based on a-priori knowledge of their relationship and were: gender; age (years); time living in the USA (years); marital status (currently married or not); education (completion of primary school, high school, college, graduate school); income (monthly income $600–$1500, $1500–$3500, >$3500); work status (working in past 3 months); and frequency of consumption of red meat, soda (soft drink) or sweetened beverages, pizza and sandwiches, sweets, vegetables, and fruits (never/hardly ever, 1–4 days per week, 5+ days per week). Based on literature regarding overweight/obesity in Brazilians, interactions between gender and marriage, work, income, or education were tested via creation of interaction terms, which were included in the multivariable logistic model. Prior published analyses of depression in this sample have been published [20]; thus, depression status was not included in this analysis. All analyses were completed in STATA version 14.0 (Stata Corp.; College Station, TX, USA).

## Results

Of the 520 initial participants in the survey, 8 pregnant women were excluded from the analysis, leaving 512 participants. An additional 151 participants were excluded due to missing data; 69 of these were missing height or weight information. The final sample eligible for this analysis included 361 Brazilian immigrants (170 men, 191 women) living in MA.

Sociodemographic characteristics for the final sample are presented in Table 1. Men made up slightly less than half of the sample (47.1%). On average, participants had lived in the USA for 12.7 years, and respondents’ average age was 39.5 years (ranging from 18 to 74 years). Approximately 45% of respondents had completed a university or graduate degree, and nearly 95% had worked in the 3 months prior to the survey. Almost half of the sample was overweight or obese (47.6%). Dietary patterns of survey participants are presented in Table 2. Normal weight and overweight or obese participants differed significantly in terms of gender and mean time living in the USA, as well as red meat and soda (soft drink) or sugary beverage consumption.

**Table 1:** Socio-demographic characteristics of Brazilian immigrant sample, 2014

Characteristic	Total Sample^a^ N (% of total)	Under/Normal Weight^b^ (BMI^c^ < 25) N (% of column)	Overweight or Obese^d^ (BMI ≥ 25) N (% of column)	*P*-Value
Overweight or Obese	172 (47.6)	–	–	–
Gender				
Men	170 (47.1)	68 (36.0)	102 (59.3)	< 0.001
Women	191 (52.9)	121 (64.0)	70 (40.7)	< 0.001
Mean Age, years	39.5	38.7	40.3	0.143
Mean Years Living in USA (SD)	12.7 (6.7)	11.6 (6.2)	13.9 (7.1)	0.001
Currently Married	208 (57.6)	107 (56.6)	101 (58.7)	0.686
Education				
Primary School	61 (16.9)	25 (13.2)	36 (20.9)	0.051
High School	137 (38.0)	72 (38.1)	65 (37.8)	0.953
University Degree	122 (33.8)	65 (34.4)	57 (33.1)	0.802
Graduate Degree	41 (11.4)	27 (14.3)	14 (8.1)	0.067
Worked in Past 3 Months	341 (94.5)	181 (95.8)	160 (93.0)	0.256
Monthly Income				
$600–$1500	103 (28.5)	54 (28.6)	49 (28.5)	0.310
$1500–$3500	162 (44.9)	89 (47.1)	73 (42.4)	0.375
> $3500	96 (26.6)	46 (24.3)	50 (29.1)	0.986

^a^Total sample size = 361

^b^Under/normal weight sample = 189

^c^*BMI* Body mass index

^d^Overweight or obese sample = 172

**Table 2:** Dietary characteristics of Brazilian immigrant sample, 2014

Food / Frequency of Consumption	Total Sample^a^ N (% of total)	Under/Normal Weight^b^ (BMI^c^ < 25) N (% of column)	Overweight or Obese^d^ (BMI ≥ 25) N (% of column)	*P*-Value
Red Meat				
Never-Hardly Ever	50 (13.9)	32 (16.9)	18 (10.5)	0.076
1–4 Days per Week	242 (67.0)	133 (70.4)	109 (63.4)	0.158
5 Days per Week- Everyday	69 (19.1)	24 (12.7)	45 (26.2)	0.001
Pizza and Sandwich				
Never-Hardly Ever	167 (46.2)	81 (42.9)	86 (50.00)	0.174
1–4 Days per Week	180 (49.9)	101 (53.4)	79 (45.9)	0.154
5 Days per Week-Everyday	14 (3.9)	7 (3.7)	7 (4.1)	0.857
Sweets (Cookies, Cakes, Candy, etc.)				
Never-Hardly Ever	101 (28.0)	56 (29.6)	45 (26.2)	0.464
1–4 Days per Week	189 (52.4)	90 (47.6)	99 (57.6)	0.059
5 Days per Week-Everyday	71 (19.7)	43 (22.8)	28 (16.3)	0.122
Soda^e^/Sugary Beverage				
Never-Hardly Ever	195 (54.0)	115 (60.9)	80 (46.5)	0.006
1–4 Days per Week	128 (35.5)	59 (31.2)	69 (40.1)	0.078
5 Days per Week-Everyday	38 (10.5)	15 (7.9)	23 (13.4)	0.093
Fruit				
Never-Hardly Ever	26 (7.2)	10 (5.3)	16 (9.3)	0.141
1–4 Days per Week	187 (51.8)	98 (51.9)	89 (51.7)	0.984
5 Days per Week-Everyday	148 (41.0)	81 (42.9)	67 (39.0)	0.451
Vegetable				
Never-Hardly Ever	6 (1.7)	2 (1.1)	4 (2.3)	0.347
1–4 Days per Week	134 (37.1)	69 (36.5)	65 (37.8)	0.801
5 Days per Week-Everyday	221 (61.2)	118 (62.4)	103 (59.9)	0.619

^a^Total sample size = 361

^b^Under/Normal weight sample = 189

^c^*BMI* Body mass index

^d^Overweight or obese sample = 172

^e^Soda refers to soft drinks

**Fig. 1:**
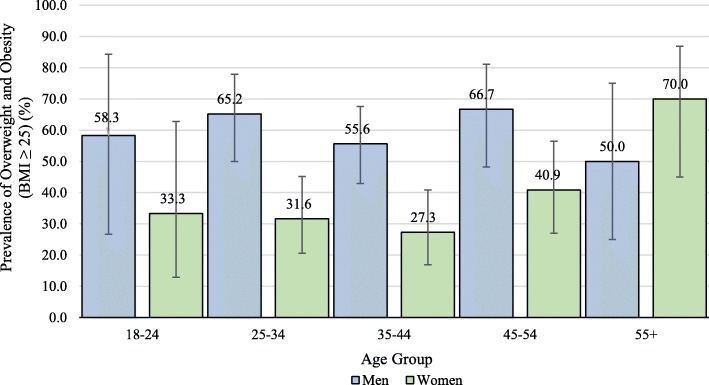
Prevalence of overweight and obesity by age group and gender in Brazilian immigrant sample, 2014. Legend: Bars represent 95% confidence intervals; BMI = body mass index

**Fig. 2:**
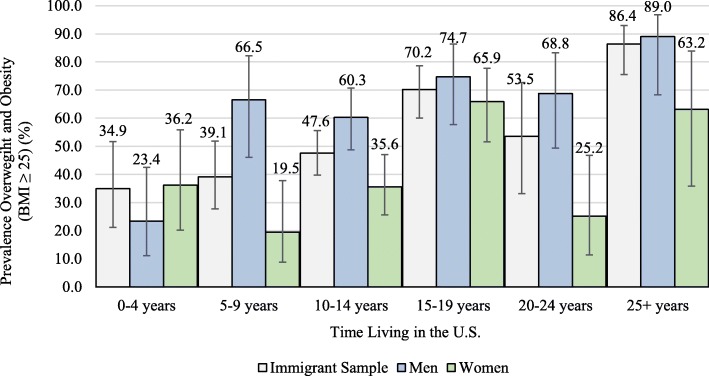
Age-standardized prevalence of overweight and obesity by time lived in the USA and gender in Brazilian immigrant sample, 2014. Legend: prevalence age-standardized to immigrant population age distribution; bars represent 95% confidence intervals; BMI = body mass index

The overall prevalence of obesity in this sample was 47.6%. When considered in different subgroups of the Brazilian immigrant population, however, prevalence varied. Among men in the sample, prevalence of overweight and obesity was 60.0%, while just 36.7% of women were overweight and obese. Prevalence also differed by age group, as shown in Fig. 1, with the highest prevalence noted in the 55–64-year-old age group (70.0%, women), and the lowest prevalence in the 35–44-year-old age group (27.3%, women). Age-standardized prevalence generally increased with 5-year increments living in the USA. However, this relationship differed between men and women (see Fig. 2). For men, age-standardized prevalence was low (23.4%) in those living in the USA for 0–4 years and then increased dramatically with each 5 more years of living in the USA. In contrast, the lowest prevalence of overweight and obesity in women was in those living in the USA 5–9 years (19.5%), with a peak in those having lived in the USA for 15–19 years. Of note, age-adjusted estimates stratified by gender and time spent in the USA contained sparse strata, with corresponding increases in uncertainty.

**Fig. 3:**
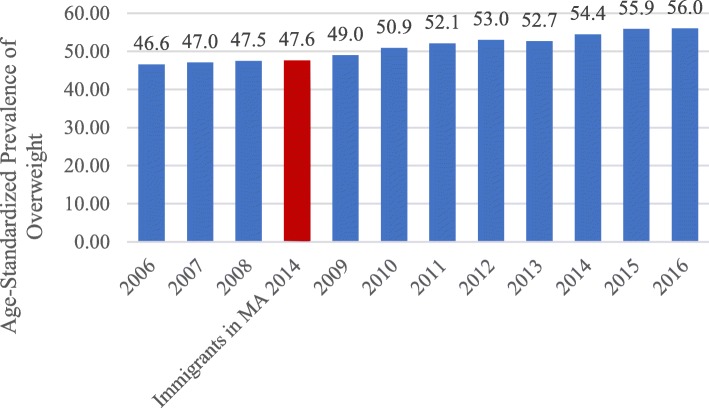
Age-standardized prevalence of overweight and obesity from Brazilian immigrant sample and Vigitel series from urban Brazil (age 18–65+), 2006–2016. Legend: prevalence age-standardized to immigrant population age distribution, immigrant sample prevalence shown in red; BMI = body mass inde

Comparisons of age-adjusted prevalence of overweight and obesity in the sample with national levels for the USA (from the 2014 NHIS) and Brazil (from the 2014 Vigitel) revealed that the immigrant population had a lower prevalence of overweight and obesity than either of the national estimates. Urban Brazil’s prevalence of overweight and obesity was 54.4%, and the USA had a prevalence of 62.0% in 2014 (compared with the Brazilian immigrant sample’s 47.6% prevalence). Additionally, when considering the series of available Vigitel data (annually conducted since 2006), Brazilian immigrants living in MA had an age-adjusted prevalence of overweight and obesity most closely resembling urban Brazil in 2008 (see Fig. 3).

Results of a multivariable adjusted logistic regression identified significant predictors of overweight and obesity in this population (see Table 3). Men had 2.30 times the odds of overweight and obesity compared with women (OR 2.30, 95% CI: 1.10, 3.78). Each additional year of living in the USA added 6% to the odds of overweight and obesity in the sample (OR = 1.06, 95% CI: 1.02, 1.11). Those who had worked in the 3 months prior to the survey had 2.9 times the odds of overweight and obesity compared with those who had not worked (OR = 2.90, 95% CI: 1.01, 8.30). Marital status, education, and income made no significant difference in the odds of overweight and obesity in this population.

**Table 3:** Multi-variable logistic regression of overweight and obesity on selected covariates

Covariate	OR	*P*-Value	95% CI
Gender			
Women	Ref	–	–
Men	2.30	0.001	(1.40–3.78)
Age	1.00	0.771	(0.98–1.03)
Time Living in the USA	1.06	0.005	(1.02–1.11)
Currently Married	1.05	0.848	(0.64–1.71)
Education			
Primary School	Ref	–	–
High School	0.64	0.188	(0.33–1.24)
University Degree	0.91	0.795	(0.44–1.86)
Graduate Degree	0.46	0.105	(0.18–1.18)
Worked in Past 3 Months	2.90	0.047	(1.01–8.30)
Monthly Income			
$600–$1500	Ref	–	–
$1500–$3500	0.95	0.867	(0.54–1.67)
>s $3500	1.03	0.932	(0.53–2.00)
Red Meat Consumption			
Never-Hardly Ever	Ref	–	–
1–4 Days per Week	1.67	0.165	(0.87–3.49)
5 Days per Week-Everyday	3.70	0.005	(1.47–9.25)
Pizza and Sandwich Consumption			
Never-Hardly Ever	Ref	–	–
1–4 Days per Week	0.49	0.012	(0.28–0.86)
5 Days per Week-Everyday	0.59	0.444	(0.15–2.29)
Sweets (Cookies, Cakes, Candy, etc.)			
Never-Hardly Ever	Ref	–	–
1–4 Days per Week	1.55	0.125	(0.88–2.73)
5 Days per Week-Everyday	0.96	0.916	(0.44–2.05)
Soda/Sugary Beverage Consumption			
Never-Hardly Ever	Ref	–	–
1–4 Days per Week	1.60	0.109	(0.90–2.85)
5 Days per Week-Everyday	2.40	0.050	(1.00–5.78)
Fruit Consumption			
Never-Hardly Ever	Ref	–	–
1–4 Days per Week	0.50	0.160	(0.19–1.32)
5 Days per Week-Everyday	0.50	0.170	(0.18–1.35)
Vegetable Consumption			
Never-Hardly Ever	Ref	–	–
1–4 Days per Week	0.38	0.334	(0.05–2.68)
5 Days per Week-Everyday	0.45	0.425	(0.06–3.17)

Frequent consumption of red meat was a strong predictor of overweight and obesity. Those who typically consumed 5 or more servings of red meat per week had 3.7 times the odds of overweight and obesity than those who never consume red meat (OR = 3.70, 95% CI: 1.47, 9.26). Regular consumption of soda (soft drink) or other sugar-sweetened drinks was also harmful, as 5 or more servings of soda (soft drink) per week was associated with 2.4 times the odds of overweight and obesity (OR = 2.40, 95% CI: 1.00, 5.78). Other dietary factors were not significantly associated with overweight and obesity.

No significant interactions were detected between gender and marital status, work status in the past 3 months or education.

## Discussion

This analysis describes the prevalence of overweight or obesity in the Brazilian immigrant population living in MA, demonstrating that this population has lower age-adjusted prevalence than national estimates for both its native and immigration destination countries. Brazilians living in MA most closely resemble urban Brazilians from 2008 in terms of age-adjusted overweight and obesity prevalence. Duration of residence in the USA is a significant predictor of overweight and obesity, with odds of overweight and obesity increasing 6% with each additional year of residence after controlling for age and other relevant covariates. Men and those who have worked in the past 3 months have greater odds of overweight and obesity than women or those who have not worked in the past 3 months. Finally, frequent consumption of red meat and soda (soft drink) or other sugary beverages are associated with higher odds of overweight and obesity.

Findings of increasing prevalence and odds of overweight and obesity with greater duration of residence in the USA is consistent with the current literature on immigrant overweight and obesity in this country, for Latinos as well as other immigrant groups [12, 27]. The lower prevalence of overweight and obesity in the Brazilian immigrant population than national estimates for both Brazil and the USA coincides with the well-described “healthy immigrant” phenomenon. The consensus, however, is that the health advantage immigrants possess fades over time, with weight gain and increased rates of overweight and obesity observed in immigrants who have lived in the USA for longer periods [4, 5, 28]; findings from this study are in agreement with this consensus.

Another explanation for the increase in overweight and obesity and general worsening of health observed in immigrant populations of all countries of origin is acculturation, or unhealthy assimilation, to poor dietary patterns common in the USA [4, 6, 27]. Data on dietary changes since moving to the USA were not included in this analysis, due to frequent missing data (> 20% for some foods) and concerns about recall bias, given the long mean duration of living in the USA (12.7 years). However, a greater number of survey participants included in this analysis responded to a question regarding physical activity change, with more than half of participants (*n* = 194, 53.7%) reporting they perform less physical activity in the USA than when they lived in Brazil. Unhealthy assimilation of health behaviors may be one explanation for increased odds of overweight and obesity with each year lived in the USA. Dietary predictors of overweight and obesity in this population include frequent (≥5 servings per week) consumption of red meat and sweetened beverages; frequent consumption of sweetened beverages has been positively correlated with overweight in immigrant and other populations in the USA [10]. Challenges immigrants face with regards to legal immigration status, employment, income generation, food and housing security, and other social determinants of health may also contribute to overweight and obesity status with time lived in the USA, though these are outside the scope of the present study or analysis.

There were some important limitations to consider with the results of this analysis. First, all data analyzed were self-reported. Particularly with self-reported weight and height, there is evidence that weight tends to be underestimated relative to measured weight (especially by women), with the opposite being true for height (especially by men) [29, 30], resulting in underestimation of BMI. This is an important consideration for the prevalence of overweight and obesity in this immigrant population. However, this should not affect the prevalence comparisons in this analysis, as Vigitel data from Brazil and the NHIS from the USA also used self-reported height and weight.

Next, it is important to note that the survey was administered with convenience sampling, and thus these results may not be generalizable to all Brazilians living in MA. Sampling the majority of immigrants at the Consulate General in Boston sought to obtain a more representative sample than religious gatherings, given the wide range of services provided at the Consulate, but representativeness of the statewide population of Brazilian immigrants cannot be guaranteed. Nevertheless, a comparison of the demographics of Brazilian immigrants living in MA from the 2014 ACS (a random sample survey conducted through the U.S. Census Bureau) with those in this survey suggests that our sample does not differ significantly from this population in terms of age, gender, English proficiency, or time living in the USA [31]. Our sample did include a higher proportion of individuals who were apparently healthy, currently married, currently employed, or had completed a graduate degree than the Brazilian-born population in the 2014 ACS. Particularities of MA as a state (such as high levels of insurance coverage) [32] may make this sample not representative of Brazilian immigrants nationally. While physical activity levels are a recognized determinant of overweight and obesity, measures of physical activity were not included in this analysis due to significant inconsistency in reporting in terms of activity frequency. Finally, the complete case analysis approach could lead to biased estimates if missing data were not missing at random. Our results indicated that missing covariate data were not associated with the main outcome of interest.

This analysis presents novel estimates of the prevalence of overweight/obesity and associated predictors in Brazilian immigrants living in MA. The positive finding that this population has a lower prevalence of overweight and obesity than its native counterparts in the USA or Brazil supports the “healthy immigrant” phenomenon observed in other literature [3–5]. Given the increasing odds of overweight and obesity in this population with duration of stay in the USA, targeted efforts should be made with this population to avoid unhealthy assimilation to USA culture. Particularly, the population should be encouraged to decrease red meat and soda (soft drink) consumption, and men and those who work should be of particular focus in these efforts.

Dissemination of these messages could potentially be spearheaded through the Consulate General of Brazil in Boston, MA or the religious communities used for sampling for this study; churches have been found to be effective settings for delivery of evidence-based obesity treatment interventions for Latino adults in the USA [33]. Other local organizations that work with immigrant communities, and Brazilians in particular, could be particularly useful hosts for intervention. The Brazilian Immigration Center/Brazilian Worker Center, the Massachusetts Alliance of Portuguese Speakers, and the Brazilian Women’s Group all have regular gatherings and offices throughout the state that provide health, employment, legal, immigration, and other services to the Brazilian immigrant population. A systematic review of obesity prevention intervention studies for immigrants found that interventions that had positive effects for obesity had a cultural focus, incorporated a participatory approach, and engaged the community via implementation in existing community structures, like those in MA [34]. Local Brazilian radio stations found around the state (Radio Brazuca, WSRO Framingham/Boston, or Transameria Hitz Boston) could promote these community-based interventions. Interventions targeted to this population should be led by community members who are fluent in Portuguese; engaging those of Brazilian descent could increase engagement of immigrants in the interventions and potentially increase their effectiveness [33]. Finally, the literature on obesity prevention and treatment interventions for Brazilian immigrants (and immigrants in general) is relatively scarce. There is an increasing number of immigrants in the USA, including the Brazilian immigrant population, who play an important role in the state of MA. Identifying additional effective, evidence-based health interventions in this population should be a priority for future research.

## Data Availability

The datasets used and/or analyzed during the current study are available from the corresponding author on reasonable request.

## Abbreviations

ACS: American Community Survey
BMI: Body Mass Index
CI: Confidence Intervals
MA: Massachusetts
NHIS: National Health Interview Survey
OR: Odds Ratio
USA: United States of America
Vigitel: Vigilância de Fatores de Risco e Proteção para Doenças Crônicas por Inquérito Telefônico
